# Impact of Diet on Gut Microbiota Composition and Microbiota-Associated Functions in Heart Failure: A Systematic Review of In Vivo Animal Studies

**DOI:** 10.3390/metabo12121271

**Published:** 2022-12-15

**Authors:** Marta Palombaro, Pauline Raoul, Marco Cintoni, Emanuele Rinninella, Gabriele Pulcini, Nadia Aspromonte, Gianluca Ianiro, Antonio Gasbarrini, Maria Cristina Mele

**Affiliations:** 1 UOC di Nutrizione Clinica, Dipartimento di Scienze Mediche e Chirurgiche, Fondazione Policlinico Universitario A. Gemelli IRCCS, Largo A. Gemelli 8, 00168 Rome, Italy; 2 Dipartimento di Medicina e Chirurgia Traslazionale, Università Cattolica del Sacro Cuore, 00168 Rome, Italy; 3 UOS Scompenso Cardiaco, Dipartimento di Scienze Cardiovascolari, Fondazione Policlinico Universitario A. Gemelli IRCCS, Largo A. Gemelli 8, 00168 Rome, Italy; 4 Gastroenterology Unit, Dipartimento di Scienze Mediche e Chirurgiche, Fondazione Policlinico Universitario A. Gemelli IRCCS, Largo A. Gemelli 8, 00168 Rome, Italy; 5 UOC Medicina Interna e Gastroenterologia, Dipartimento di Scienze Mediche e Chirurgiche, Fondazione Policlinico Universitario A. Gemelli IRCCS, Largo A. Gemelli 8, 00168 Rome, Italy

**Keywords:** heart failure, diet, gut microbiota, TMAO, short-chain fatty acids, gut metabolites, cardiac hypertrophy, fiber, choline

## Abstract

Heart failure (HF) represents a cardiovascular disease with high mortality and morbidity. The latest evidence shows that changes in the composition of the gut microbiota might play a pivotal role in the prevention and management of HF. This systematic review aims at assessing the potential associations between the diet, gut microbiota, and derived metabolites with the outcomes of HF. A systematic literature search was performed up to July 2022 on the PubMed, Web of Science, and Scopus databases. The PRISMA guidelines were followed when possible. The risk of bias was assessed with the SYRCLE and ARRIVE tools. A total of nine pre-clinical studies on animal models, with considerable heterogeneity in dietary interventions, were included. High-fiber/prebiotic diets (*n* = 4) and a diet rich in polyphenols (*n* = 1) modified the gut microbiota composition and increased microbial metabolites’ activities, linked with an improvement in HF outcomes, such as a reduction in systolic blood pressure, cardiac hypertrophy, and left ventricular thickness. A high-fat diet (*n* = 2) or a diet rich in choline (*n* = 2) induced an increase in TMAO and indole derivative production associated with a decrease in cardiac function, systemic endotoxemia, and inflammation and an increase in cardiac fibrosis and cardiac remodeling. Although results are retrieved from animal studies, this systematic review shows the key role of the diet—especially a high-fiber and prebiotic diet—on gut microbial metabolites in improving HF outcomes. Further studies on human cohorts are needed to identify personalized therapeutic dietary interventions to improve cardiometabolic health.

## 1. Introduction

The gut microbiota represents the most abundant community of microorganisms living in the human gastrointestinal tract [1]. Indeed, more than 1000 different bacterial species, with a few dominant phyla—Bacteroidetes, Firmicutes, Actinobacteria, and Proteobacteria—regulate essential host functions such as the maintenance of intestinal barrier integrity, nutrient absorption, and immunity homeostasis [2]. In humans, Firmicutes and Bacteroidetes are the most dominant phyla, with more than 90% of the total bacteria [3]. At the genus and species levels, the taxa diversity and microbial richness, as well as the microbiome functions, characterize the gut microbial signature, which is unique for each individual [4]. Throughout human life, several environmental factors (infancy factors, use of medication, diet, socioeconomic factors, etc.) influence the bidirectional relationships between the gut microbiota (in terms of composition and functions) and human health and diseases [5].

Dysbiosis, which is defined as an alteration of gut microbiota composition and function, seems to play an important role in the pathogenesis and progression of cardiovascular diseases, including heart failure (HF) [6,7,8,9,10,11,12,13,14]. HF is a complex clinical syndrome with symptoms and signs caused by structural and/or functional cardiac abnormality [15], representing a major clinical and public health problem. Indeed, worldwide, around 64.3 million people have been diagnosed with HF [16,17]. Underlying causes of HF can be various, and often, in an individual, it is not possible to identify a single major factor, because, especially in the chronic setting, multiple causes co-exist [18]. HF is characterized by typical anatomical alterations resulting in gut edema, congestion, and impaired intestinal permeability [19]. This leads to intestinal bacterial translocation, immune activation, and consequently low-grade systemic inflammation that adversely affects the course of the disease [20]. In the last year, microbial sequencing approaches and computational analyses have highlighted the key role of the gut microbiota and its derived metabolites in the host regulation of both inflammatory and fibrotic responses and cardiac remodeling [21,22]. Moreover, microbial metabolites, including short-chain fatty acids (SCFA), trimethylamine N-oxide (TMAO), lipopolysaccharide (LPS), and others, may exert different essential effects on the incidence and development of HF [23].

SCFAs include acetate, propionate, and butyrate, which are produced through the gut bacterial fermentation of dietary fiber [24]. TMAO is a metabolite produced through the action of gut microbial trimethylamine (TMA) lyase from choline—or carnitine—or betaine-rich foods contained in egg yolk, red meat, and certain seafoods [25]. Some in vitro studies support that TMAO promotes vascular inflammation and is associated with enhanced atherosclerosis and thrombosis [26,27]. In a study from Savi et al., it was demonstrated that TMAO affected myocardial contractility, interfering with calcium dynamics [28]. LPS are endotoxins primarily found in the outer layer of Escherichia genera, which are associated with increased host inflammatory responses [29]. Recently, the therapeutic potential of the gut microbiota and its microbial metabolites has been demonstrated in HF through different approaches, such as dietary/prebiotic supplementation [30]. Prebiotics are nutrients that induce the growth or activity of beneficial gut bacteria in the host [31]. The number of studies on the role of the gut microbiota in the pathogenesis of HF is growing.

In this context, we chose to conduct a systematic review to assess the impact of dietary interventions on gut microbiota composition and microbiota-associated functions in HF.

## 2. Methods

This systematic review followed the Preferred Reporting Items for Systematic Reviews and Meta-Analyses (PRISMA) guidelines [32]. The PRISMA checklist is detailed in Table S1.

### 2.1. Inclusion Criteria

The inclusion criteria are presented according to the PICOS criteria (Table 1).

The exclusion criteria were the following: (1) non-English articles; (2) in vitro and human models; (3) review articles; (4) not fulfilling the PICOS criteria.

### 2.2. Data Sources and Search Strategy

The search was carried out using three electronic databases, MEDLINE (via PubMed), ISI Web of Science, and Scopus. Multiple search terms were used, including diet, food fiber, ketogenic, vegetarian, mediterranean, vegan, low protein diet, plant-based diet, low phosphate diet, high sulfur amino acid diet, high pral diet, alkalin diet, western diet, gluten free, protein free, high protein diet, gut microbiota, microflora, gut bacteria, microbiome, SCFA, butyrate, acetate, proprionate, TMAO, trimethylamine N-oxide indole, phenol indoxyl sulfate indole, acetic acid, LPS, lipolysaccharide, gut barrier. The search string for each database is described in Table S2. Hand searching of eligible studies was done to identify studies that may not have been found in the databases.

### 2.3. Study Selection

Titles and abstracts were screened for eligibility based on inclusion criteria. Two reviewers independently selected studies. All titles assessed as ineligible were excluded. Disagreements were resolved by consensus between these two authors and, if necessary, discussed by two additional authors.

### 2.4. Data Extraction and Reporting

After full-text analysis, the following information was extracted from the included articles: first author, year of publication, country, animal type, age, sample size, diet intervention type, duration of diet intervention, significant compositional changes in gut microbiota (compared with control groups), significant effects on microbiota-associated functions (compared with control group), significant effects on heart failure outcomes. Data were reported using an Excel© (Microsoft Office, Redmond, WA, USA) spreadsheet specifically developed for this study. Each full-text article was retrieved, and any ineligible articles were excluded from the reasoning reported. Differences in judgment between two reviewers were settled by discussion and consensus.

### 2.5. Quality Assessment

The SYRCLE’s risk of bias tool for animal studies [33] and the ARRIVE tool [34] were used to assess the risk of bias. The risks of bias data were extracted by four different authors and all disagreements were resolved by consensus by the remaining authors.

## 3. Results

### 3.1. Study Selection

**Figure 1:**
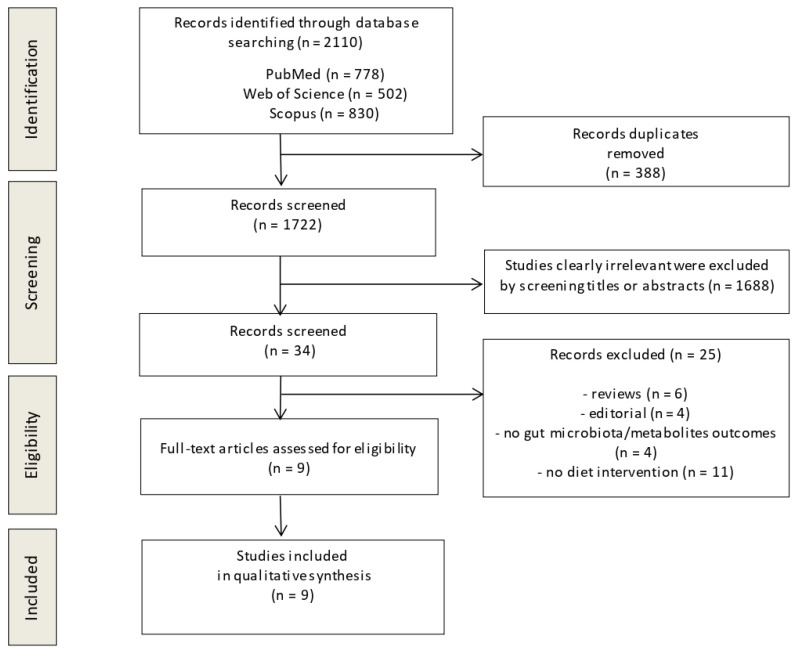
Preferred Reporting Items for Systematic Reviews and Meta-Analyses (PRISMA) flow diagram.

The flow diagram in Figure 1 displays the results of the literature search and study selection process. A total of 2110 studies were initially identified. After duplicate removal, 1722 studies remained for title and abstract screening. Thirty-four studies were selected and, after full-text analysis, twenty-five studies were excluded for the following reasons: review (*n* = 6), editorial (*n* = 4), no gut microbiota outcomes (*n* = 4), no diet intervention (*n* = 11). Nine studies [35,36,37,38,39,40,41,42,43] were identified for inclusion in the systematic review.

### 3.2. Study Characteristics

Nine studies were included: three from China [36,42,43], two from the USA [39,40], two from Australia [35,38], one from the United Kingdom [41], and one from Russia [37]. Nutritional interventions differed across studies and included a high-fiber diet/prebiotic intervention (*n* = 4) [35,36,37], choline diet (*n* = 2) [39,40], Western diet (*n* = 1) [42], obesogenic/high-fat diet (*n* = 1) [41], and polyphenol supplementation (*n* = 1) [43]. Seven studies were mice models [35,36,38,39,40,41,42] and two were rat models [37,43]. The date of publication ranged from 2016 [39] to 2021 [43] and the sample size from 18 [36] to 121 [40]. The duration of the intervention varied from 7 days [37] to 20 weeks [40]. Five studies analyzed the compositional changes in the gut microbiota [35,36,37,38,41,43]. All studies assessed microbiota-derived metabolite variations. Heart failure outcomes varied across studies: blood pressure (*n* = 3), cardiac hypertrophy (*n* = 3), ventricular thickness (*n* = 3), left ventricular ejection fraction (*n* = 2), BNP (*n* = 2), interstitial fibrosis (*n* = 4), cardiac remodeling (*n* = 3). Table 2 summarizes the characteristics of the included studies.

### 3.3. Results

Table 2 details the results of included studies by dietary approach.

#### 3.3.1. Impact of Diet Rich in Fiber on Gut Microbiota Composition and Functions in HF

A total of three studies [35,36,37] showed the effect of a high-fiber diet on the gut microbiota and HF outcomes.

Jama et al. showed, irrespective of mice type (wild-type or mice with dilated cardiomyopathy), significant compositional variations in mice receiving a high-fiber diet (for 7 weeks) compared with a control diet, with a significant increase in *Bacteroidetes* and *Bacteriodales* abundance [35]. Zhang et al. found, in healthy mice fed with a high-fat diet + oral *Lycium Barbarum* (100 mg/kg once a day for 2 months), an increase in the abundance of *Gordonibacter*, Parabacteroides, *Anaerostipes*, *Blautia*, *Hungatella*, and *Marvin bryantia* compared with mice fed with a high-fat diet or healthy mice [36]. In rats with HF fed with fermented wheat bran and inactivated Saccharomyces cerevisiae culture for 7 days, a reduction in the levels of *Pretovella* spp., *Fusobacterium* spp., *Helicobacter pylori*, *Lactobacillus* spp., *Enterococcus* spp., and *Actinomyces* and an increase in the levels of *Bifidobacterium* spp., *Propionibacterium* spp., and *Eubacterium* spp. were observed compared with rats with HF fed a control diet [37].

All three studies found a positive effect of a high-fiber diet on gut microbial activities in HF mice or rats. There was a significant expansion in splenic T reg cells in mice with dilated cardiomyopathy fed with a high-fiber diet compared with wild-type mice (*p* = 0.009) [35]. Zhang et al. assessed, in healthy mice fed with a high-fat diet + oral *Lycium Barbarum* (100 mg/kg once a day for 2 months), a reduction in intestinal permeability and an increase in indole derivatives (indole-3-acrylic acid, methyl indole-3-acetate) compared with mice fed with a high-fat diet or healthy mice [36].

Two studies demonstrated the beneficial effect of a high-fiber diet and SCFA supplementation on cardiac function and remodeling [36,38]. Zhang et al. reported noticeable improvements in respect to systolic function and diastolic relaxation, as well as cardiac remodeling [36]. Similar results were obtained by Marquez et al., who demonstrated that a high-fiber diet and SCFA supplementation can counteract cardiac alteration induced by DOCA surgery in mice [38]. In particular, in the intervention groups, authors observed reduced blood pressure, limited cardiac hypertrophy, and lower ventricular chamber dilatation [38]. On the other hand, Jama et al. showed no significant changes in cardiac hypertrophy, lung congestion, and blood pressure in response either to a high-fiber diet or acetate supplementation in model mice with severe dilatated cardiomyopathy [35].

#### 3.3.2. Impact of Choline Diet on Gut Microbiota Composition and Functions in HF

Two studies analyzed the effects of a choline diet and of a diet supplemented with choline metabolite TMAO on the gut microbiota metabolites and heart function [39,40]. In both studies, HF was induced in mice surgically using transverse aortic constriction (TAC). Analysis of plasma from mice was performed, respectively, at 12 weeks [39] and 17 weeks [40] after TAC. Both studies showed that plasma levels of TMAO were significantly higher when animals were fed with a diet containing 0.12% TMAO added to the standard rodent chow compared with controls [39,40]. Interestingly, it was evidenced that, even after TMAO withdrawal, at 6 weeks, plasma levels of this metabolite remained elevated compared with controls [40]. Moreover, in mice fed with TMAO, a significant increase in plasma betaine levels was observed [39]. A diet containing 1 or 1.2% choline equally determined a significant rise in TMAO levels in the blood, demonstrating that choline is effectively converted into TMAO by intestinal microbes [39,40]. The use of iodomethylcholine, a small molecule that inhibits the gut microbial conversion of choline, given orally, has been demonstrated to be effective in avoiding the elevation of circulating TMAO [40].

In both trials, cardiac function was evaluated via echocardiography at 3-week intervals following baseline echocardiography studies at 1 week before TAC surgery for 12 [39] and 17 weeks [40]. Circulating BNP was measured at the end of the observation period in each group as an indicator of HF severity. Moreover, to further evaluate cardiac remodeling, histological and immunochemical analysis was performed to detect and quantify fibrosis [39,40]. Myocardial and pulmonary tissue weight were measured to investigate cardiac enlargement and pulmonary edema as the main consequences of HF [39,40].

The TMAO and choline group experienced a significant worsening of cardiac function in comparison with the control diet. Notably, in these experimental arms, echocardiography showed an increased LV dimension, abnormal IVSd, poorer LVFS, and lower LVEF [39,40]. Importantly, it was observed that TMAO withdrawal, as well as the use of iodomethylcoline, attenuated the pathological actions of the dietary intervention and all measured endpoints improved [40]. Similarly, BNP levels significantly increased in intervention groups and decreased after TMAO withdrawal or with the administration of a choline trimethylamine lyase inhibitor [39,40].

In one study, after 12 weeks of TMAO or choline diet, enhanced collagen and fibrosis were observed in the hearts of mice [39]. In the other study, although dietary TMAO did not directly affect cardiac fibrosis, it was associated with the increased expression of profibrotic genes in the heart [40]. An increase in total kidney fibrosis was observed in both intervention groups [39,40].

In one study, cardiac enlargement and pulmonary edema were evident from the direct observation of hearts taken from mice fed with TMAO and choline [39], but these results were not fully confirmed in a later study [40].

#### 3.3.3. Impact of Western/Obesogenic Diet on Gut Microbiota Composition and Functions in HF

Two studies reported the consequences of high calories and a high-fat diet on microbiota and HF [41,42]. Kein et al. investigated the changes in microbial composition in young and aging mice after two months of nutrition with a calorie-dense obesogenic diet (OBD): a single fatty-acid-enriched diet (10% safflower oil) that mimicked a standard Western diet [41].

Microbiome analysis of fecal samples demonstrated important differences in gut microbiome composition between mice fed with STD versus OBD. In both young and old mice, the consumption of OBD was associated with an increase in actinobacteria, and the genus *Allobactulum* was the most represented [41]. On the other hand, *Bacteroides* S24-7 was the most abundant in mice fed with STD [41].

It was observed that mice fed with WD for 8 weeks had significantly higher levels of plasma TMAO compared with the control diet. The use of DMB, an inhibitor of trimethylamine formation, reduced TMAO levels not only in WD-fed mice but also in the control group [42]. Microbiome composition seemed to influence systemic inflammation. Indeed, OBD-fed young mice revealed an increased neutrophil to lymphocyte ratio [41]. As for aging mice, both the STD and the OBD group showed an elevation in neutrophil populations, reflecting the fact that age is the most influential factor [41]. Additionally, OBD impacted splenic leukocytes, resulting in a reduction in immune-responsive F4/80+ and CD169+ macrophages in aging mice [41]. Moreover, the blood dosage of isoprostanoids was indicative of increased lipid peroxidation and oxidative stress in OBD-fed mice regardless of age [41].

Only one of the two studies directly analyzed the effects of WD on cardiac function [42]. In this study, echocardiography was performed at baseline and after 8 weeks of diet, showing that WD correlated with poorer cardiac function [42]. The authors evidenced a decrease in LVEF (around 19%) and increases in LVICT, LVIRT, and MPI. DMP has been able to prevent the development of these alterations [42].

Through histological analysis, it was observed that interstitial fibrosis was remarkably increased in the hearts of mice fed with WD relative to controls [42]. Moreover, WD was associated with decreased levels of anti-inflammatory cytokines (IL-10) and elevated levels of pro-inflammatory cytokines (TNFα, IL β), which promote cardiac fibrosis [42].

#### 3.3.4. Impact of Polyphenols on Gut Microbiota Composition and Functions in HF

One study observed the potential benefits of a diet containing polyphenols on HF, in particular analyzing doxorubicin-induced heart failure. The authors evaluated a four-week treatment with purified polyphenols from *Arctium Iappa* L. (ALPP) at the dosages of 50 mg/kg (ALPP1) and 150 mg/kg (ALPP2) [43].

Important variations in microbial composition were observed. In the DOX group, there was a reduction in the richness and alpha diversity of the gut microbiota, as shown by the reductions in the Ace, Chao, and Shannon indexes [43]. Stool samples from mice with DOX-induced heart failure indicated an abundance of Proteobacteria and reduction in Firmicutes, with the upregulation of the Bacteroide/Firmicutes ratio. Notably, ALPP acted as a modulator of microbiota and could reverse the alteration induced by HF [43]. The heat map showed no significant difference between the control and ALPP groups. On the contrary, in the DOX group, a loss of some beneficial bacteria was observed (for example, *Roseburi*a and *Lactobacillus*), together with the proliferation of some pathogens (such as *Enterococcus* and *Escherichia*, *Shigella*) [43].

Changes in microbial composition induced by HF and polyphenols clearly influenced the formation of specific gut microbiota metabolites. In the DOX group, there was high production of TMAO compared with the ALPP + DOX group [43]. ALPP treatment promoted the production of short-chain fatty acids (SCFAs), which positively modulate the immune system and inflammatory response and are essential for the health of colonocytes and to preserve the integrity of the gut barrier.

Polyphenols could counteract DOX-induced changes in the heart. Indeed, pretreatment with ALPP counteracted the loss of body weight and decreased the heart and kidney indices in a dose-dependent manner [43]. The histological analysis showed that heart tissue in HF was characterized by the infiltration of inflammatory cells, hypertrophy of myocardiocytes, and focal areas of necrosis. Interestingly, ALPP reduced these pathological lesions, as well as NO and pro-inflammatory cytokine secretion [43]. Moreover, serum CK and LDH were elevated in DOX-induced HF as signs of myocardial ischemia, but treatment with polyphenols has been effective in reducing the levels of these two markers, with a clearly dose-dependent effect [43].

### 3.4. Quality Assessment

Tables S3 and S4 summarize the quality assessment results.

The ARRIVE guidelines were used to assess the quality of the nine included animal studies. All included animal articles adequately provided an accurate title and abstract, a structured and thorough introduction, an ethical statement for mammalian studies, and an adequate study design, except for one study [37], where the ethical statement was not indicated. None of the studies calculated the sample size; thus, a too small number of experimental animals may lead to a non-significant statistical analysis. All studies described statistical methods. Baseline characteristics (body weight, age, and gender) at baseline were reported in all studies. All studies reported outcomes in terms of analyzed numbers, adverse events, interpretation, and generalizability.

The SCYRCLE tool was also used to assess the risk of bias of the included studies. As regards sequence generation, in eight studies, the animals were randomly distributed into different groups, except for one study [37]. Indeed, Vlasov et al. did not specify how animals were allocated to different groups. Regarding allocation concealment, the concealment was not clear for all studies, except for one study [40]. Indeed, Organ et al. explicated the concealed procedure and blinded the investigators to randomization until the end of the study. All included studies had an unclear risk of performance bias. Indeed, the animals were not randomly housed but, in most of the studies, animals were placed in a single cage. Moreover, in all studies, it was not clear whether the investigators selected animals at random for outcome assessment. As regards data and outcome reporting, the risk of bias was low for all studies, since the outcome data reported in each study were completed for each outcome. Finally, the studies did not report other limitations, resulting in an unclear risk of bias for other sources of bias.

Overall, the quality of each study could be improved due to the unclear randomization of housing, unclear blinding outcome assessment, and absence of sample calculation. However, the animal population for all studies was homogenous. All studies had a control group with similar characteristics to the experimental groups. The reporting of outcomes (complete outcome data reporting, adequate outcome reporting) was acceptable for all studies.

## 4. Discussion

This systematic review highlighted the gut-modulatory activity of different dietary interventions in animals with HF. All included animal studies demonstrated a complex association between diet intervention, gut microbiota composition and functions, and HF outcomes. Thus, as one of the important modulators in the gut microbiota, a high-fiber diet, prebiotic diet, and diet rich in polyphenols positively modify the microbial composition (increase in gut microbial diversity and richness) and microbial metabolite activity (reduction in endotoxemia, reduction in intestinal permeability, increase in SCFA production), which were linked with an improvement in HF outcomes, such as a reduction in systolic blood pressure, cardiac hypertrophy, and left ventricular thickness [36,37,38,43]. On the contrary, Western diets such as a high-fat diet or diet rich in choline negatively modify the gut microbial composition, inducing an increase in TMAO and indole derivative production, a decrease in cardiac function, systemic endotoxemia, inflammation, gut barrier dysfunction, and an increase in cardiac fibrosis and cardiac remodeling [36,39,40,41,42].

It is well known that fiber has a prebiotic effect that selectively promotes intestinal eubiosis. Indeed, several studies have demonstrated that HF is associated with an altered gut microbiota and that these changes can influence disease progression in both animals and humans [44,45,46]. Modifications of the gut microbiome are strictly linked to HF due to the anatomical disorders that occur at the level of the gut barrier [47]. Thus, a high-fiber diet can be a powerful tool in HF therapy and prevention. In line with our results, Dietary Approaches to Stop Hypertension (DASH) and the Mediterranean diet appeared to be promising strategies for the prevention of HF in humans. The potential benefit is linked to the fact that these diets are rich in fiber and micronutrients (such as polyphenols and other antioxidants).

Polyphenols are compounds, naturally found in fruits, vegetables, and cereals, that have antioxidant properties. We know that dietary polyphenols exert an improving effect on several cardiovascular risk factors, such as blood pressure, endothelial function, and plasma lipids [48]. One of the well-known polyphenols is resveratrol, largely found in grapes. Studies on mice suggested that this compound may improve cardiac remodeling, diastolic cardiac function, and cardiac glucose metabolism in an HF mice model [49]. Moreover, it may increase exercise capacity, counteracting fatigue, which is clearly one of the main signs of HF [49]. What emerged from our review is that polyphenols are important modulators of the microbiota. They can influence the production of SCFAs, with a beneficial effect on the gut barrier and systemic inflammation [43].

One of the most-studied gut microbiota metabolites in HF is TMAO. It was demonstrated that TMAO levels led to a higher long-term mortality risk in HF patients [50]. The levels of this metabolite rely on the gut microbiota composition and diet content. Choline, betaine, and L-carnitine are the main precursors of TMAO. Chronic consumption of red meat increases plasma TMAO levels, while a vegan diet is effective in reducing its levels [51,52]. The studies reported in this review highlighted the fact that a diet rich in choline is responsible for a significant elevation in TMAO levels in mice, and this leads, as a consequence, to the impairment of cardiac function [39,40]. A similar alteration occurred with the consumption of a Western diet, rich in saturated fat and sucrose [42]. Thus, an obesogenic diet and Western diet appear to be detrimental in this setting [39,40,42].

Other types of dietary approaches should be evaluated, such as dietary carbohydrate restriction [53]. In a recent study, a low-carbohydrate diet reduced the progression of pathological hypertrophy in HF through ketone body production and the inhibition of underlying signaling mechanisms, such as mTOR [54]. Moreover, some evidence supported the fact that, in HF, the failing heart progressively loses its capacity for the use of fatty acids and glucose oxidation as an energy source and shifts to ketone bodies as a fuel source for ATP production [55]. In this context, ketosis may have a therapeutic role, even if results are limited and myocardial ketone use seems to be only adaptative [56]. Notably, a short-term ketogenic diet (KD) has a beneficial effect on glucose control and weight loss and thus can be effective in reducing cardiovascular risk. However, a recent animal study showed an increase in inflammation-inducing *Enterobacteria* and a reduction in SCFA levels in the cecum in a carbohydrate-restricted diet group compared with a control group [57]. Even in humans, KD seemed to negatively affect the intestinal microbiota [58]. Thus, the effect of a low-carbohydrates diet in preventing or reversing the progression of heart failure remains to be clarified and the gut microbiota should be analyzed.

*Helicobacter pylori* (*H. pylori*) is one of the most-studied bacteria in the gastrointestinal tract. Chronic infection is linked to peptic ulcer disease and gastric cancer and it is responsible for a state of persistent inflammation that is associated with other extra-intestinal diseases. In the literature, many studies have highlighted the connection between *H. pylori* positivity and cardiovascular risk [59,60]. Indeed, the role of *H. pylori* infection in HF patients can be of interest.

This systematic review has some limitations. First, no human studies assessing the associations between diet intervention, gut microbiota, and HF outcomes were found. Secondly, although all included studies enrolled rodent models, the animal sample size, the type of diet intervention, and their exposure duration differed between studies and this does not allow clear results. Thus, although all studies found a significant impact on gut microbiota and HF outcomes, it remains difficult to reach firm conclusions. Further animal homogeneous studies are needed to evaluate the impact of different dietary patterns as promising therapeutic approaches. The impact of a high-fiber diet and prebiotic diet on the gut microbiota in animals and humans with HF should be further evaluated. However, the results of the studies analyzed in this review suggest that HF is often associated with an altered gut microbiota composition, with a prevalence of *Enterococcus* and *Clostridium* spp., a reduction in the abundance of *Bifidobacterium* spp., and thus an increased Firmicutes/Bacteroidetes ratio (F/B). Indeed, a Mediterranean diet rich in fiber and antioxidants, and low in saturated fat and animal protein, can be of great benefit in HF patients.

As a conclusion, this systematic review suggests a strong correlation between HF and gut microbiota metabolites, and specific associations between diet interventions, microbiota, and gut microbiota metabolites and progression. Although these results need to be confirmed by larger studies, they aim at considering diet strategies toward regulating the gut microbiome and using its metabolic pathways to treat HF, potentially improving prognostic outcomes.

## Figures and Tables

**Table 1:** PICOS criteria for inclusion of studies.

Population	Animals or Humans
Intervention	Diet interventions (dietary factors or dietary patterns or prebiotic intake)
Comparator	Any comparator
Gut microbial outcomes	Differences in alpha diversity (Chao1 index, Shannon diversity index, Simpson diversity index) and beta diversity of fecal microbiota at the end of the intervention
	Differences in abundance of bacterial taxa
	Differences in fecal SCFAs, muc-2 gene expression, TMAO levels, indole levels, phenol indoxyl sulfate, indole acetic acid levels, LPS levels
Heart failure outcomes	Blood pressure (in mmHg) Cardiac hypertrophy (in mm) Ventricular thickness (in mm) Left ventricular ejection fraction (%) BNP (in pg/mL) Interstitial fibrosis (%) Cardiac remodeling
Study design	All types

Abbreviations: BNP, brain natriuretic peptide; SCFA, short-chain fatty acid; LPS, lipopolysaccharide; TMAO, trimethylamine N-oxide.

**Table 2:** Characteristics of included animal studies (listed by diet intervention).

First Author, Year of Publication, Country	Animal Type	Sample	Dietary Intervention Type	Duration of Dietary Intervention	Methods of Characterization of Microbiota and/or Metabolites	Changes in Gut Microbiota (Compared with Control Groups)	Effects on Microbiota-Associated Functions (Compared with Control Group)	Effects on Heart Failure Outcomes
Fiber diet								
Jama, 2020, Australia [35]	C57BL/6 male mice ^1^	N = 48 7–8 mice/group 2 groups DCM mice WT mice	3 interventions High-RS diet (High-fiber SF11-025, Specialty Feeds) Control diet with acetate supplementation (magnesium acetate, Sigma-Aldrich, 200 mM in drinking water) Control chow diet	7 weeks	16S rRNA amplicon sequencing	Irrespective of mice type: ● significant compositional variations in high-RS diet vs. control diet ● ↑ *Bacteriodetes* and Bacteriodales abundance in mice following high-RS diet vs. control diet	Significant expansion of splenic T regulatory (Treg) cells in DCM mice fed a high-RS diet (*p* = 0.009) vs. WT mice. and a non-significant increase compared to DCM mice on the control diet (*p* = 0.05)	In high-RS diet or acetate supplementation vs. control diet, in DCM mice, no significant improvements in: ● cardiac hypertrophy ● cardiac remodeling ● systolic and diastolic pressure
Zhang, 2020, China [36]	Healthy specific-pathogen-free C57BL/6J male mice^2^	N = 18 6/group	Control diet HFD HFD + oral *Lycium barbarum* polysaccharide 100 mg/kg once a day (HFPD)	2 months	16S rRNA amplicon sequencing	● ↑ *Bacteroides, Muribaculum, Alistipes, Parasuterella*, and *Alloprevotella* abundance in control group vs. HFD diet groups. ● ↑ *Lactobacillus, Bifidobacterium, Enterococcus, Lactococcus, Romboutsia* in the HFD group vs. control group ● ↑ Gordonibacter, Parabacteroides, *Anaerostipes, Blautia, Hungatella, Marvin bryantia* abundance in the HFPD group vs. other groups.	● ↑ the indole derivatives (indole-3-acrylic acid, methyl indole-3-acetate, and DI-indole-3-lactic acid) in HFPD vs. HFD group ● ↑ intestinal permeability in the HFD fed mice vs. control ● ↓ intestinal permeability in HFPD group	● In the HFD group, depressed left ventricular systolic function and abnormal diastolic relaxation ● In the HFD group vs. the control and HFPD groups, ↑ cardiac remodeling (↑ LVPWd, LVPWs LVAWd,…, LVAWs, and ↓ LVIDd, LVIDs, LVEDd, LVEDs, EF, and FS)
Vlasov, 2020, Russia [37]	Female adult rats	N = 30 *n* = 10 female rats/group 3 groups - HF female rats - control healthy - control group HF female rats	Pre-treatment with prebiotic complex (fermented wheat bran and inactivated *Saccharomyces cerevisiae* culture) Control	7 days	Not specified	HF rats vs. controls ● ↑ *Prevotella spp*., *Fusobacterium* spp., *Kingella* spp., *Enterococcus* spp., *Clostridium* spp., and *Lactobacillus* spp. abundance ● ↓ *Bifidobacterium* spp., *Propionibacterium* spp., and *Eubacterium* spp. abundance HF rats pretreated with prebiotic complex vs. HF rats ● ↓ *Pretovella* spp., *Fusobacterium* spp., *Helicobacter pylori*, *Lactobacillus* spp., *Enterococcus* spp., *Actinomyces* abundance ● ↑*Bifidobacterium* spp., *Propionibacterium* spp., and *Eubacterium* spp. abundance	HF rats vs. controls ● ↑ endotoxemia LPS levels (*p* = 0.03) HF pretreated with prebiotic complex vs. HF rats ● ↓ endotoxemia LPS levels (*p* = 0.02)	
Marques, 2017, Australia [38]	C57Bl/6 male mice ^3^ (hypertension induced by treatment with uni- nephrectomy and implantation of deoxycorticosteroid acetate or sham pellets)	N = 64 *n*= 6–15 mice/group 6 groups - sham + control -DOCA + control -DOCA + fiber -DOCA + acetate - sham + fiber -sham + acetate	Control High-fiber diet (72,7% fiber) SCFA supplementation (200 mmol/L magnesium acetate)	6 weeks	16S rRNA amplicon sequencing	● Significant compositional variations in mice fed a control diet vs. high-fiber diet (in both sham and DOCA groups) ● ↑ acetate-producing bacteria in mice fed with high-fiber diet ● ↓ Firmicutes to Bacteroidetes ratio (F/B) in mice fed with high-fiber diet or acetate ● ↑ levels of *Bacteroides acidifaciens* spp. in mice fed a high-fiber diet or acetate vs. mice fed the control chow		In high-fiber diet and acetate supplementation groups: ● ↓ systolic blood pressure ● ↓ cardiac hypertrophy ● ↓ left ventricular wall thickness ● ↓ left ventricular chamber dilatation In mice fed with acetate supplementation: ● ↓ renal fibrosis
Choline diet								
Organ, 2016, USA [39]	C57BL6/J male mice ^4^ (cardiac pressure overload and HF were induced using transverse aortic constriction TAC surgery)	N = 36 *n* = 10–12 mice/group	Control diet (TD.130104) Diet containing 0.12% TMAO added to the standard rodent chow (TD.07865) Diet containing 1.2% choline added to the standard rodent chow (TD.09041)	15 weeks	Stable isotope dilution LC/MS/MS for quantification of the total choline, TMA, TMAO, and betaine levels		● ↑ TMAO levels in mice fed either TMAO or choline vs. mice fed a control diet ● ↑ plasma betaine levels in the mice fed TMAO vs. mice fed a control diet	In mice fed either TMAO or choline vs. mice fed a control diet: ● ↓ cardiac function ● ↑ left ventricular end-systolic diameter and end-diastolic diameter ● ↓ IVSd ● ↓ left ventricular ejection fraction.supplemented diet: ● ↑ heart weight and ↑ left atrial weight ● ↑ lung weight /tibia length ● ↑ BNP levels ● ↑ interstitial and perivascular fibrosis
Organ, 2020, USA [40]	C57BL/6 male mice ^4^ (cardiac pressure overload and HF were induced using transverse aortic constriction TAC surgery)	N= 121 N= 19–35 mice/group	Control diet Diet supplemented with 0.12% TMAO (Subgroup withdrawal of dietary TMAO at 6 weeks after TAC surgery) Diet supplemented with 1% choline Diet containing 1% choline + 0.06% iodomethylcholine (choline TMA lyase inhibitor)	20 weeks	Nexera ultra-high-performance liquid chromatography system for quantification of plasma TMAO levels		● ↑ plasma TMAO in mice fed with TMAO supplemented diet; withdrawal of dietary TMAO significantly reduced plasma levels, but they remained elevated compared with control diet ● ↑ circulating TMAO in choline group; TMAO remained at control levels in choline + iodomethylcholine group	In the TMAO group compared with control: ● ↑ adverse cardiac remodeling (↑ LVESD, ↑ LVEDD) (adverse cardiac remodeling was attenuated when TMAO was withdrawn) ● ↑ BNP levels (TAMO withdrawal led to reduced circulating BNP) ↑ heart weight (no significant reduction after TMAO withdrawal) ● no difference in cardiac fibrosis but ↑ levels of profibrotic genes ↑TGFβ, ↓COL1A1, ↓TIMP2 after TMAO withdrawn ● ↑ renal fibrosis (no significant difference after TMAO withdrawal) Choline diet vs. control and choline diet + iodomethylcholine: ● ↑ adverse cardiac remodeling (↓LVFS) ● ↑ BNP levels ● no significant changes in heart weight ●↑ levels of cardiac profibrotic genes (↑TGFβ, ↑ MMPs) ● ↑ renal fibrosis
Kain, 2019, United Kingdom [41]	Male C57BL/6J mice ^5^	*n* = 3–8 mice/group Young mice Aging mice	Calorie-dense obesogenic diet (OBD) 10% safflower oil Control diet 4% safflower oil diet	2 months	6S variable region 4 rRNA gene DNA sequencing and Quantitative Insights Into Microbial Ecology informatics	● ↑ *Allobaculumin* genus in young and aging mice fed with OBD versus control diet ● ↑ Actinobacteria in OBD group, irrespective of age ● OBD in aging mice disrupted the composition of the gut microbiome	● OBD dysregulation of splenic leukocytes with ↓ immune-responsive F4/80 + and CD169 + macrophages in aging mice	OBD in aging dysregulated splenic leukocytes with the expansion of systemic inflammation and the beginning of the incomplete resolution of inflammation in acute HF
Chen, 2017, China [42]	Male CD1 mice ^2^	N = 40 10 mice/group Mice with DMB Mice without DMB (inhibitor of TMA formation)	Western diet (2% total fat, 12.8% saturated fat, and 30% sucrose) Control diet (17% total fat, 0.8% saturated fat, and 0% sucrose)	8 weeks	Liquid chromatography coupled with triple-quadrupole mass spectrometry		Compared with mice fed a control diet, in mice fed a WD: ● ↑ TMAO levels in mice fed with WD vs. mice fed with control (*p* < 0.05)	in WD vs. control diet: ↓ cardiac function ● ↓ LVEF ● ↑ LVICT ● ↑ LVIRT ● ↑ MPI DMB prevented WD-induced changes ● ↑ cardiac fibrosis DMB prevented fibrosis ● ↑ pro-inflammatory cytokines (TNFα; IL 1β) DMB determined increase in anti-inflammatory cytokines
Wu, 2021, China [43]	BALB/C male mice ^1^	N = 60 2 groups HF rats Control rats	Polyphenols from *Arctium lappa* L. (ALPP) containing 16 phenolic substances Control (saline solution) ALPP1 (50 mg/kg) ALPP2 (150 mg/kg)	1 month	Gut microbiome: 16S rRNA amplicon sequencing SCFAs levels: Gas chromatography–mass spectrometry	● ↓ Shannon, ACE, and Chao1 indices in HF group vs. control group, ALPP2, and ALPP2 + HF ● ↓ number of OTUs, ↓ bacterial richness, ↑ Proteobacteria, ↓ Firmicutes in HF group vs. control group, ALPP2, and ALPP2 + HF ● ↓ number of *Roseburia, Lactobacillus*, *Lachnospsiraceae, Prevotellaceae, Ruminococcaceae, Erysipelotrichaceae*, in HF group fed with control diet vs. control ● ↑ number of *Bilophila*, *Enterococcus, Erysipeloclostridium, Escherichia* and *Shigella*, in HF group fed with control diet vs. control ● no significant changes in all microbial flora at the genus level (ratio Bacteroidetes/Firmicutes), Firmicutes, Proteobacteria, and Bacteroidetes in control group vs. ALPP2 control and ALPP2 HF group (remission effect)	● ↑ SCFA levels in the ALPP2 group vs. control group ● ↓ SCFA levels in the HF group vs. control group ● ↑ SCFA levels in the ALPP2 HF group vs. HF group	

Abbreviations: COL 1A1 collagen type I alpha 1; DCM, dilated cardiomyopathy; DMB, 3.3-dimethyl-butanol; DOCA, deoxycorticosteroid acetate; EF, endocardial fractional shortening; FS, midwall fractional shortening; HF, heart failure; HFD, high-fat diet; IVSd, interventricular septal wall thickness; LVAWd, left ventricular anterior wall diastolic thickness; LVAWs, left ventricular anterior wall systolic thickness; LVEDd, left ventricular diastolic diameter; LVEDs, left ventricular systolic diameter; LVESD, left ventricular end systolic diameter; LVEDD, left ventricular end diastolic diameter; LVFS, left ventricular fractional shortening; LVICT, left ventricular isovolumetric contraction time; LVIDd, left ventricular diastolic dimension; LVIDs, left ventricular systolic dimension; LVIRT, left ventricular isovolumetric relaxation time; LVPWd, left ventricular posterior wall diastolic thickness; LVPWs, left ventricular posterior wall systolic thickness; MMPs, matrix metalloproteinase; MPI, myocardial performance index; RS, resistant starch; SCFA, short-chain fatty acid; vs., versus; TGF-β, transforming growth factor beta; WT, wild type; ↑, increase; ↓ decrease. Footnotes: ^1^ 4-week-old; ^2^ 8-week-old; ^3^ 6-week-old; ^4^ 10–12- week-old; ^5^ young mice (2-month-old) and aging (18-month-old).

## Supplementary Materials

The following supporting information can be downloaded at: https://www.mdpi.com/article/10.3390/metabo12121271/s1, Table S1: PRISMA checklist; Table S2: Search strategies; Table S3: Quality assessment results with ARRIVE guidelines; Table S4: Quality assessment results with SYRCLE tool.
